# Novel Cellulose Derivatives Containing Metal (Cu, Fe, Ni) Oxide Nanoparticles as Eco-Friendly Corrosion Inhibitors for C-Steel in Acidic Chloride Solutions

**DOI:** 10.3390/molecules26227006

**Published:** 2021-11-19

**Authors:** Mohamed Gouda, Hany M. Abd El-Lateef

**Affiliations:** 1 Chemistry Department, College of Science, King Faisal University, Al-Ahsa 31982, Saudi Arabia; 2 Chemistry Department, Faculty of Science, Sohag University, Sohag 82524, Egypt

**Keywords:** cellulose derivatives, corrosion inhibitors, metal oxide nanoparticles, cellulose nanocomposites, Electrochemical Impedance Spectroscopy, surface morphology

## Abstract

Novel environmentally-friendly corrosion inhibitors based on primary aminated modified cellulose (PAC) containing nano-oxide of some metals (MONPs), for instance iron oxide nanoparticles (Fe_3_O_4_NPs), copper oxide nanoparticles (CuONPs), and nickel oxide nanoparticles (NiONPs), were successfully synthesized. The as-prepared PAC/MONPs nanocomposites were categorized using Fourier transform infrared spectroscopy (FT-IR), transmission electron microscope (TEM), field-emission scanning electron microscopy (FE-SEM), energy-dispersive X-ray spectroscopy (EDX), X-ray diffraction (XRD), and selected area diffraction pattern (SAED) techniques. The data from spectroscopy indicated that successful formation of PAC/MONPs nanocomposites, as well as the TEM images, declared the synthesized PAC/Fe_3_O_4_NPs, PAC/CuONPs, and PAC/NiONPs with regular distribution with particle size diameters of 10, 23 and 43 nm, respectively. The protection performance of the as-prepared PAC and PAC/MONPs nanocomposites on the corrosion of C-steel in molar HCl was studied by the electrochemical and weight-loss approaches. The outcomes confirmed that the protection power increased with a rise in the [inhibitor]. The protection efficiency reached 88.1, 93.2, 96.1 and 98.6% with 250 ppm of PAC/CuONP, PAC/Fe_3_O_4_NPs, and PAC/NiONPs, respectively. PAC and all PAC/MONPs nanocomposites worked as mixed-kind inhibitors and their adsorption on the C-steel interface followed the isotherm Langmuir model. The findings were reinforced by FT-IR, FE-SEM and EDX analyses.

## 1. Introduction

Corrosion is a dangerous phenomenon devastatingly affecting mechanical and modern applications, especially in the oil and gas enterprises. Consequently, regulatory metal decomposition is a significant action of specialized, affordable, ecological, and appealing significance to spare tremendous costs in resources, hardware, and construction. The utilization of corrosion inhibitors is perhaps the most ideal choice for governing the corrosion of metals in different harsh media. Through the utilization of little particle organic and inorganic corrosion inhibitors for different issues, the utilization of corrosion inhibitors based on polymers came into the spotlight [1]. Polymers are materials that have fantastic adhesive properties on metal surfaces. A wide scope of polymers have been identified for their corrosion resistant properties as both pre-covering [2] on the metal or just as an inhibitor in an assortment of corrosive liquids [3,4,5,6,7,8,9,10]. Furthermore, the use of carbohydrate polymers in corrosion hindrance gave a course to utilize materials that are biodegradable, artificially steady, eco-friendly with the one-of-a-kind repressing property, low cost, and renewable [11,12]. The molecular weights decide the quality of carbohydrate polymers as inhibitors, the nearness of adsorption focuses, accessibility of security shaping gatherings, and the nearness of cyclic rings in the class of the carbohydrate [13]. The corrosion restraining exercises of these polymers are ascribed to their capacity to frame edifices with metal particles on the outside of metals through their practical gathering. The complex is framed to cover the outside of the metals because of their enormous surface region, subsequently shielding the metals from destructive species present in their condition [14]. This inhibitive action relies upon the cyclic rings and heteroatoms (nitrogen and oxygen) present in the structure of the polymer. In addition, the possible utilization of carbohydrate polymers has been assessed in an expansive range of fields, for example, medication conveyance [15], adsorption [16], wastewater treatment [17,18], consumption obstruction [19], and catalysis [20]. Cellulose is the amongst the carbohydrate polymers and is sustainable by practically limitless crude material and particularly encouraging as it delivers great mechanical execution with likely biocompatibility, non-harmfulness, biodegradability, and the existence of reactive gatherings. This last element is engaging, as its additional modification would be possibly helpful when used to include another polymer, or adjust their properties which will offer new and capricious applications [21].

Moreover, cellulose is the most bountiful water-insoluble natural polysaccharide. Semi water-insoluble cellulose derivatives are engineered simply of cellulose. The corrosion inhibitor properties of cellulose derivatives were identified for mild steel in various acid solutions [7,8,9]. Bayol et al. have considered the adsorptive conduct of cellulose derivatives on mild steel in HCl solution [7]. Umoren et al. revealed the hindrance capability of cellulose derivatives for sulphuric acid corrosion of steel [8,9], and the impacts of synergism and enmity of halide ions with cellulose derivatives on the corrosion restraint. The electrochemical properties of meager films of two subsidiaries of the biopolymer, hydroxypropyl methylcellulose, specifically hydroxypropyl methylcellulose phthalate and hydroxypropyl methylcellulose acetate succinate, in an emphatically acidic condition were examined. The corrosion resistance of hydroxypropyl methylcellulose-subordinate covered steel was assessed utilizing electrochemical impedance spectroscopic estimations and potentiodynamic polarization [22].

It is reported that for organic materials, for example, polymers or macromolecules, having utilitarian gatherings (-OH, -COOH, -NH_2_, and so on), are seen as anti-corrosive materials in various destructive media [8,9,23,24,25,26,27,28,29,30,31,32]. Bigger corrosion hindrance efficiencies that have been observed utilizing polymers are not just because of the nearness of π-electrons; however, it very well may be likewise ascribed to the bigger sub-atomic size which guarantees more prominent inclusion of metallic surface [30]. Hydroxyethylcellulose is a water-solvent polymer obtained from cellulose, and is a moderately modest, non-harmful, eco-accommodating corrosion inhibitor. It has broad applications as a folio, thickener, stabilizer, suspension, and water-holding specialist in the food business, pharmaceutical, corrective, paper, and other mechanical regions [33]. Hydroxyethylcellulose has been investigated to hinder the corrosion of aluminum and steel in HCl solutions [34].

With the quick progression of nanotechnology, slight thickness films in the micrometric and nanometric measures are expanding their notoriety in logical and innovative uses [35]. Nanoscale materials have a high propensity to cooperate to frame accumulation [36]. Its one-of-a-kind properties are fundamental because of the greater surface area of the nanoscale compounds in contrast with the micro-scaled compounds brought about by its enormous surface area to volume ratio [37]. There are different reports regarding the enhancement of corrosion resistance utilizing nanoparticles, for instance, TiO_2_ [38], Cu_2_O [39], ZnO [40], ZrO_2_ nanoparticles [41], Fe_3_O_4_ [42], SiO_2_ [43] and organo-clay nanoparticles [44]. In addition, metal oxide nanoparticles are of unique enthusiasm owing to their differing structure, optical, magnetic, mechanical, thermal, and electronic features. Among the wide assortment of metal oxide nanoparticles, Taiwo et al. prepared and characterized nanocomposites of some designated polymers, namely, poly(vinylpyrrolidone), poly(ethylene glycol), and polyacrylonitrile containing ZnO, and studied their application as inhibitors for the corrosion of C-steel in hydrochloric acid solution [45].

Herein, primary aminated modified cellulose (PAC) and Fe_3_O_4_NPs, CuONP and NiONPs in PAC structure were designed and smoothly fabricated by using in situ deposition. The as-prepared materials were characterized via Fourier transform infrared spectroscopy (FT-IR), field-emission scanning electron microscopy (FE-SEM)/energy-dispersive X-ray spectroscopy (EDX), X-ray diffraction (XRD), transmission electron microscope (TEM), and selected area diffraction pattern (SAED). Moreover, we report, for the first time, the application of PAC and Fe_3_O_4_NPs, CuONP and NiONPs in PAC structure, as corrosion inhibitors for C-steel in HCl solution. Weight loss, electrochemical (PDP and EIS), and surface topology measurements (FT-IR and FE-SEM/EDX), were used to identify the anti-corrosive activity.

## 2. Experimental Procedures

### 2.1. Materials

Microcrystalline cellulose by a degree of polymerization (DP) of 10,000, nickel nitrate, copper chloride, ferric chloride, isopropyl alcohol, H_2_SO_4_ (98%), sodium borohydride, 1,2-epoxypropene (98%) and NH_4_OH (37%) were obtained from Sigma-Aldrich Co. (Saint Louis, MO, USA). The primary amine reagent was synthesized according to the reported method [46]. Further chemicals of analytical grade were used without further refinement.

The working electrodes for experiments were made of C-steel (C1018) and have an area of 0.5 cm^2^ with a chemical structure (wt%) of manganese (0.06%), carbon (0.19%), chromium (0.75%), nickel (0.06%), and iron (98.94%). The mechanical characteristics of the C-steel were measured and provided by the supplier displayed as follows: tensile strength ≈ 490 MPa and elongation to failure ≈ 16%. The findings were provided by European Corrosion Supplies Ltd. The electrode was used without any modifications. The protecting performance of the prepared nanocomposites was measured at six various doses quantified by 25, 50, 100, 150, 200, and 250 mg L^−1^.

### 2.2. Synthesis of Primary Amine (PA)

Primary amine was synthesized by adding one mole of ammonia to one mole of epichlorohydrine (ECH) in the presence of 100 mL isopropanol. The mixture was stirred at 31–34 °C for 7 h. At the end of the reaction, the excess of isopropanol was removed by a rotary evaporator at 30 °C under vacuum. Unreacted ECH was removed by five frequent washes with chloroform. 

### 2.3. Synthesis of Cellulose Containing Primary Amino Group (PAC)

PAC was synthesized according to the reported procedure [47] as follows: 1.0 mole of microcrystalline cellulose was continuously mechanical stirred at 25 °C for 5 min in the presence of 1.0 mole of sodium hydroxide. Synthesized primary amine reagent (3-chloro-2-hydroxypropyl amine) was added to the aforementioned mixture under continuous stirring for 5 min at 25 °C. The mixture was transferred to the thermostatic water at 80 °C for 180 min. At the end of the reaction duration, the synthesized samples were acidified with 1.0 N HCl solution. The prepared acidified samples were washed with an ethanol/water mixture (80:20) for 12 h at 70 °C to remove unreacted amine using a soxhlet extractor. The degree of substitution (DS) of synthesized PAC was investigated based on a measure of their nitrogen content using the Kjeldahl procedure. DS of synthesized PAC was 0.33. 

### 2.4. Synthesis of PAC/MONPs Nanocomposites

PAC containing MONPs was synthesized according to the reported route [48]. To a weighing bottle containing 100 mL of 0.5 M metal salt solution (isopropyl alcohol/water 50:50) (*v/v* %) 0.5 g PAC was added. The pH of the reaction mixture was adjusted to pH 9.5 and kept shaken at 25 °C for 24 h. At the end of the duration time, sodium borohydride (0.25 g) was added. The aforementioned reaction mixture was transferred to the ultrasonic sonication bath for 30 min with constant stirring using a mechanical stirrer for 30 min at 25 °C. The prepared samples were filtered-off, washed and dried at 80 °C for 2 h. The dried samples were transferred to the furnace at 400 °C for 8 h.

### 2.5. Physicochemical Characterization

Surface morphology of primary aminated cellulose without and with metal oxide nanoparticles was portrayed using FE-SEM supplemented with EDX (JOELF, Tokyo, Japan). Diameter and distribution, as well as the diffraction of prepared nanocomposites, were investigated by transmission electron microscopy (TEM) coupled with SAED (TEM-ZEISS-EM-10-GERMANY). The crystal pattern of the primary aminated cellulose without and with metal oxide nanoparticles was assessed utilizing X-ray diffraction with a 2*θ* extended from 10° to 80° (Rigaku, Tokyo, Japan). Furthermore, primary aminated cellulose without and with metal oxide nanoparticles was characterized utilizing FTIR spectrometer-8400S (SHIMADZU, Kyoto, Japan) to the range of 400–4000 cm^−1^. 

### 2.6. Corrosion Measurements and Experimental Setup

The corrosion experiments were performed at atmospheric pressure using an orthodox three-electrode system. The C-steel with an immersed surface area of 1.0 cm^2^ functioned as a working electrode, a Pt-mesh electrode as a counter, and a silver/silver chloride/KCl_sat_ electrode as a reference. The protective action of the nanocomposite additives on the corrosion of C-steel in hydrochloric acid medium was examined by the Gamry Galvanostat/Potentiostat/ZRA analyzer. Prior to each experiment, a steady open circuit potential (*E*_OCP_) was reached within 40 min after the specimen was dipped in the corrosive medium. EIS tests were carried out within the frequency range of 0.3 MHz to 100 kHz at the *E*_OCP_. Furthermore, PDP studies were achieved in the potential range of ±250 mV vs. *E*_OCP_ at a scan rate of 0.2 mV/s. Slope lines at the overpotentials of cathodic and anodic branches of Tafel diagrams were induced by Gamry Echem Analyst software version 5.50 [4,5]. All corrosion experiments were repeated three-times to ensure the accurateness of the experimental outcomes. The weight loss procedures were similarly performed as titled in our earlier study [3]. The corrosion tests were completed at a temperature range from 303 to 333 K. 

### 2.7. Surface Topology Investigations

The surface investigation by FE-SEM/EDX (JOELF, Tokyo, Japan) of the working electrode was attained after exposure to these specimens for 48 h in molar hydrochloric acid in the absence and existence of 250 mg/L of PAC and PAC/NiO NPs at 303 K. Additionally, the protecting layer designed at the C-steel/HCl interface containing PAC/NiO NPs inhibitor were inspected by accumulating the formed film after 48 h of dipping by scrabbling the C-steel substrates, washing them with bidistilled H_2_O, and then testing them through an FT-IR instrument.

## 3. Results and Discussion

### 3.1. Characterizations of PAC/MONPs Nanocomposites

#### 3.1.1. FTIR

**Figure 1:**
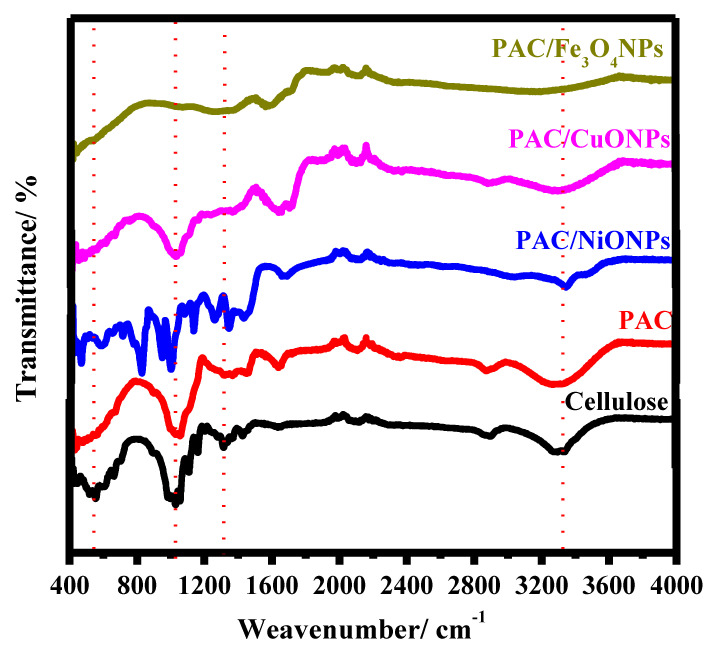
FTIR spectral analysis of cellulose, primary aminated modified cellulose (PAC), PAC/NiONPs, PAC/CuONPs and PAC/Fe_3_O_4_NPs nanocomposites.

FT-IR investigation of cellulose, PAC, PAC/NiONPs, PAC/CuONPs and PAC/Fe_3_O_4_NPs are shown in Figure 1. The bands are found in two wavenumber regions of 3681–2801 cm^−1^ and 1651–405 cm^−1^, which represent the unmodified cellulose. The distinctive beaks at 3501–3001, 2895, 1429, 1028, and 893 cm^−1^ are conforming to the vibration of -OH, -C-H-, -CH_2_ bending, -C-O-C- of pyranose ring, and the *β*(1→4)-unhydroglycosidic link, correspondingly [49]. With regard to PAC, the results showed that it is similar to unmodified cellulose, as the results showed the presence of the OH stretching with an increase in the bandwidth, which designates the presence of the -N-H. In addition, a weak band was observed with very small intensity at 1301 cm^−1^, which designates the occurrence of expansion vibrations of the C-N bond, confirming the presence of an amino substitute in cellulose [50]. Moreover, Figure 1 shows FTIR spectra PAC/MONPs, and the obtained spectra revealed that the band that concerned OH converts to low intensity in PAC/NiONPs and disappeared in PAC/CuONPs and PAC/Fe_3_O_4_NPs. Alternatively, in PAC/NiONPs, PAC/CuONPs, and PAC/Fe_3_O_4_NPs, the C-N peak appeared as a weak band with low intensity at 1345, 1375, and 1363 cm^−1^, correspondingly. Furthermore, the band regarding the pyranose ring -C-O-C- became weak and shifted in all FAC/MONPs. Additionally, weak representative bands at 457, 481, and 535 cm^−1^ are allocated to PAC/NiONPs, PAC/CuONPs, and PAC/Fe_3_O_4_NPs vibration, respectively. These results proved the formation of nMO/Acell nanocomposites successfully [51,52,53].

#### 3.1.2. XRD

**Figure 2:**
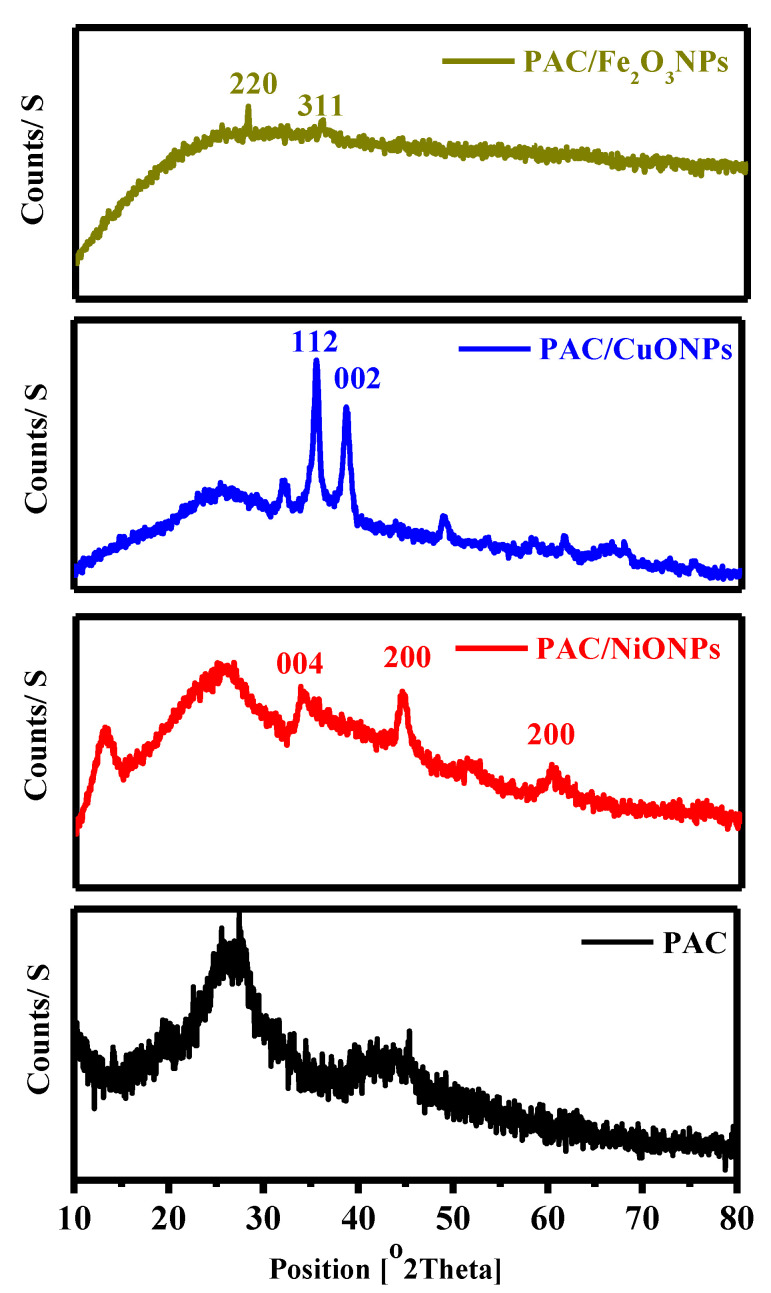
X-ray diffraction (XRD) pattern of PAC, PAC/NiONPs, PAC/CuONPs and PAC/Fe_3_O_4_NPs nanocomposites.

The crystal structure of PAC and PAC/MONPs has been studied using X-ray diffraction. The size of the PAC/MONPs crystal has been determined utilizing Scherer’s equation according to the following equation:

(1) $$D_{\mathrm{nm}}=\frac{k\lambda }{\beta \cos\theta }$$

where *D*_nm_ represents the crystal size (in nm), *k =* 0.890 represents the constant of Scherrer, *λ =* 0.15425 nm symbolizes the wavelength of X-ray, *β* characterizes the complete thickness at a partial maximum of the band (FWHM), and *θ* represents the Bragg angle [54]. Figure 2 demonstrates the crystallographic behavior of the PAC, PAC/NiONPs, PAC/CuONPs, and PAC/Fe_3_O_4_NPs, correspondingly. Crystalline bands for unmodified cellulose were detected about 2*θ* of 16.0° and 22.0°, consistent with (110) and (200) planes according to the literature [55]. In PAC, a variation in the crystallinity occurred due to the disappearance of a peak at 2*θ* of 16°, and a peak at 22° was shifted to approximately 25.8°. Therefore, PAC/MONPs display remarkable alterations in the PAC patterns. As displayed in Figure 2, the presence of NiONPs (apparently of PAC) was identified by bands at 2*θ* of 44.1° and 60.8°, which are known as peaks of cubic NiONPs crystal conforming to crystal planes (200), as well as (220), correspondingly. On the other hand, a band at 2*θ* of 33.5° is recognized as Ni_2_O_3_ (004), which is consistent with the data (JCPDS-Card 47-1049) [56]. Nevertheless, NiONPs did not change the crystal structure of PAC because the peak at 25.8° had not disappeared. Moreover, Figure 2 shows a robust and acute peak at 2*θ* of 28.1° (112) for cubic copper oxide crystallites. Correspondingly, weak bands at 2*θ* of 32°, 35.5° and 38.6° are allocated to (110), (002) and (111) crystal level surface (JCPDS-Card 033-0480) [57,58]. The PAC crystal construction crashed, and thus the peak at 25.9° was completely repelled due to a good interaction of Cu with PAC. Finally, Figure 2 displays the XRD configuration of PAC/Fe_3_O_4_ that represents feeble diffraction bands at 2*θ* of 28.6° and 35.6°, that are allocated to (220) and (311) planes [59]. The considered typical crystal size of MONPs according to Equation (1) was 9–15 nm. Meanwhile, these results were well-matched with the results obtained from SAED analysis.

#### 3.1.3. FE-SEM and EDX

**Figure 3:**
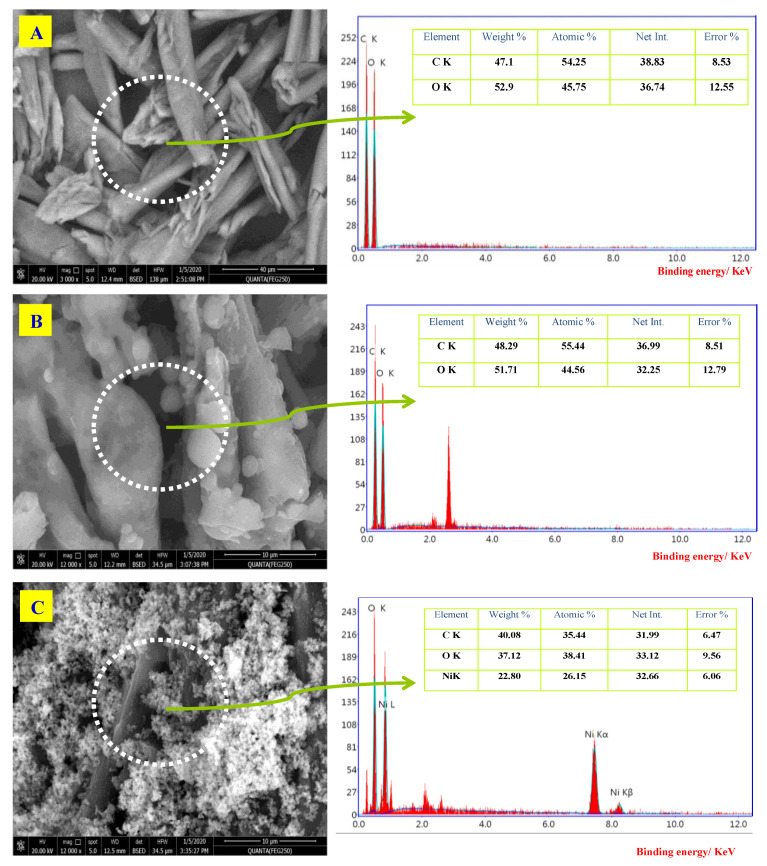

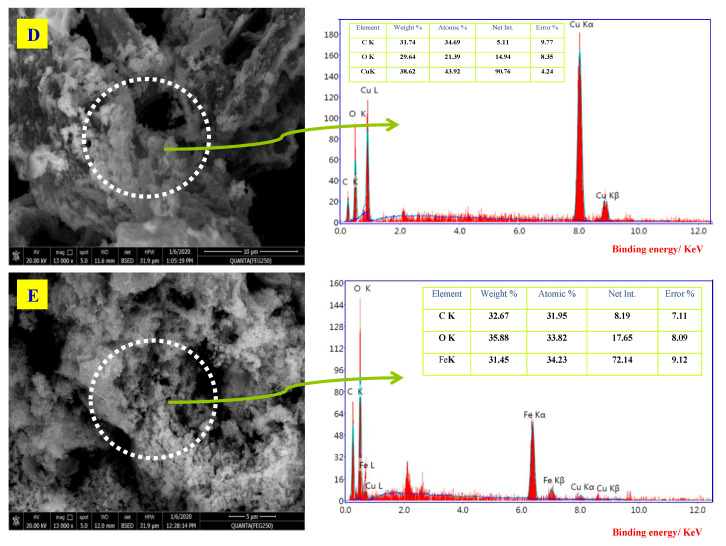
Field-emission scanning electron microscopy (FE-SEM) and energy-dispersive X-ray spectroscopy (EDX) spectra of (**A**) cellulose, (**B**) PAC, (**C**) PAC/NiONPs, (**D**) PAC/CuONPs and (**E**) PAC/Fe_3_O_4_NPs nanocomposites.

Figure 3A–D shows the scanning electron microscope and EDX for unmodified cellulose and amino cellulose samples without and with metal oxide nanoparticles. It can be seen from the SEM micrographs that unmodified cellulose surface is plain and uniform in size, plated formed, and with a strip-like structure. Furthermore, it is seen that the morphology structure of unmodified cellulose has changed after modification with the primary amine. PAC samples were unpredictably wrinkled with light spots of spongy structures. This property was apparently attributed to the presence of an amino group. In addition, Figure 3C–E shows that no agglomeration and uniform spreading of MONPs occurs inside the PAC by means of cubic form, as detected in Figure 3C–E. In conjunction with the EDX investigation, the data confirmed the formation of nanocomposites and the spectra display the occurrence of NiONPs (signals at 7.80 and 8.40), CuONPs (signals at 8.0 and 9.0 eV), Fe_3_O_4_NPs (signals at 6.20 and 7.0 eV), while no other impurity was identified in the spectrum of the sample.

#### 3.1.4. TEM and SAED Analyses 

**Figure 4:**
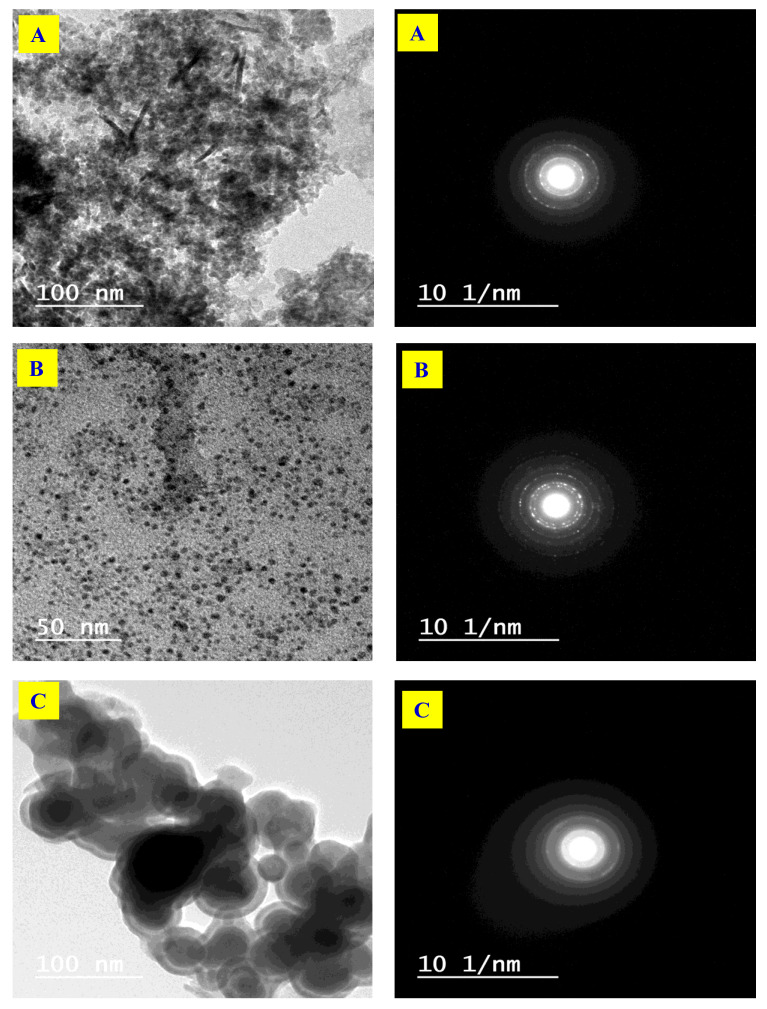
Transmission electron microscope (TEM) and selected area diffraction pattern (SAED) of (**A**) PAC/NiONPs, (**B**) PAC/CuONPs and (**C**) PAC/Fe_3_O_4_NPs nanocomposites.

TEM is a procedure utilized to break down the distribution and diameter of fabricated MONPs in PAC. Figure 4A–C shows the TEM and SAED of prepared PAC/NiONPs, PAC/CuONPs, and PAC/Fe_3_O_4_NPs, individually. TEM pictures show level and smooth films of MONPs surfaces without any conglomeration. Moreover, it is seen that MONPs showed up as round dim spots and consistently scattered inside the PAC. Besides, the size dispersion of the MONPs is generally restricted and their diameter was found around 10, 23, and 25 nm for NiONPs, CuONPs, and Fe_3_O_4_NPs, separately. Additionally, the SAED was applied to portray the qualities and structures of the MONPs existing on the PAC interface (Figure 4A–C). The chosen region for the diffraction design with the allocated Miller records has firmly coordinated the qualities acquired by XRD. The SAED pictures demonstrated the translucent highlights of the readied PAC/MONPs and the presence of the MONPs on the PAC interface, and these MONPs crystals help with dispersing the electron radiates.

### 3.2. Corrosion Protection Measurements

#### 3.2.1. Weight-Loss Investigations and Influence of Temperature 

**Figure 5:**
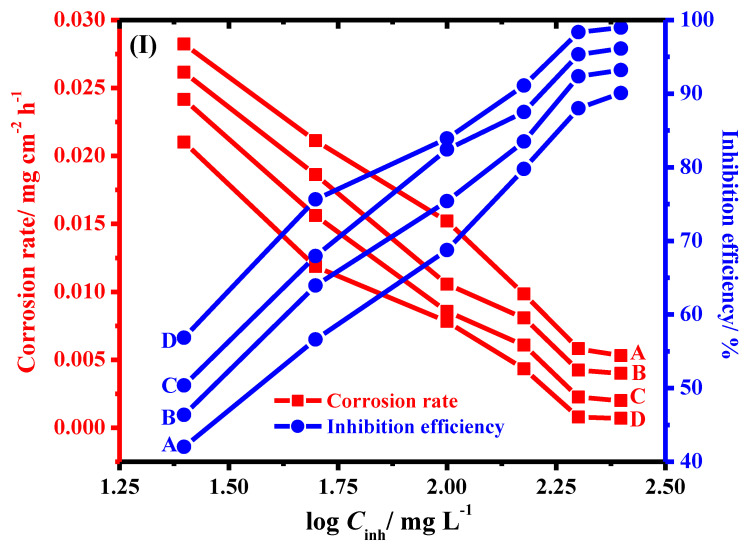
Change of corrosion rate and inhibition efficiency of C-steel in 1.0 M HCl solution with concentration of (**A**) PAC, (**B**) PAC/CuONPs/, (**C**) PAC/Fe_3_O_4_NPs and (**D**) PAC/NiONPs at 303 K.

The values of corrosion rate (CR) and inhibition capacity intended from the weight loss measurements at 303 K are presented in Figure 5. As can be observed from Figure 5, the inhibition power increases from 42.2, 46.1, 50.3 and 56.5% to 89.5, 93.4, 96.2 and 98.8 %, and the corrosion rate decreases from 0.02823, 0.02616, 0.02416 and 0.02101 mg cm^−2^ h^−1^ to 0.0053, 0.004, 0.00201 and 0.00069 mg cm^−2^ h^−1^ as the concentration of PAC, PAC/nCuO, PAC/nFe_3_O_4_ and PAC/nNiO increases from 25 to 250 ppm, respectively. An increment in the concentration of inhibitors suppressed C-steel corrosion in molar HCl solution and consequently provided higher surface coverage. Furthermore, the PAC/nNiO shows excellent corrosion protection features, which could be observed from higher inhibition power (98.8 %) and the lower corrosion rate (0.00069 mg cm^−2^ h^−1^) by comparing with that of PAC, PAC/nCuO and PAC/nFe_3_O_4_. 

**Figure 6:**
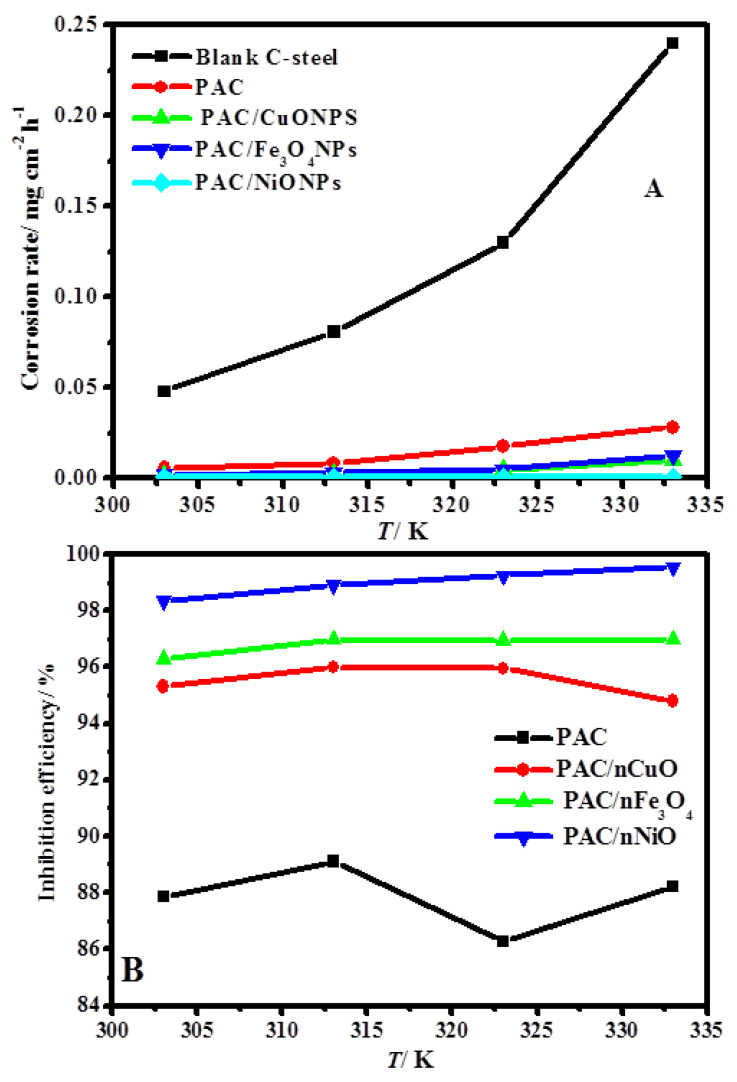
Corrosion rate (**A**) and inhibition efficiency (**B**) of C-steel in 1.0 M HCl solution containing 250 ppm of inhibitors at various temperatures.

To study the temperature impact on the protected characteristic of PAC, PAC/nCuO, PAC/nFe_3_O_4_, and PAC/nNiO inhibitors, weight loss measurements were performed at a temperature range of 30–60 °C for 48 h. Figure 6A,B show the corrosion rate (A) and protection efficiency (B) of C-steel in hydrochloric acid in the existence of 250 ppm PAC, PAC/CuONPs, PAC/Fe_3_O_4_NPs, and PAC/NiONPs inhibitors at various temperatures. The corrosion rate values of the blank and inhibited C-steel specimens in HCl solution increment when the temperature rises; however, the increased amplitude of PAC/Fe_3_O_4_NPs, and PAC/NiONPs is small. In the inhibited solutions containing PAC and PAC/nCuO inhibitors, the inhibition capacity first increases and then diminishes. The decrease in the protection power could be related to the desorption of PAC and PAC/CuONPs from the C-steel interface at a higher temperature. This phenomenon indicates that, at a higher temperature, PAC and PAC/CuONPs molecules fundamentally adsorb on the C-steel surface by physisorption [60]. On the other hand, the protection capacity of PAC/NiONPs (Figure 6B,C) increases from 98.1% to 99.3% with a rise in temperature. In addition, the protection capacity of PAC/NiONPs at a higher temperature is ~99.3%, indicating that the whole C-steel surface is nearly entirely wrapped by the protective film. This is principally related to its small size and high density of ground boundary enabling good adhesion, which allows an excellent physical coverage of the metal surface and thus higher protection capacity [61]. By examining the change of inhibition power of PAC/NiONPs with the temperature (Figure 6B), it can be concluded that chemisorption plays a significant function in the process of adsorption [62].

#### 3.2.2. Potentiodynamic Polarization Study (PDP)

**Figure 7:**
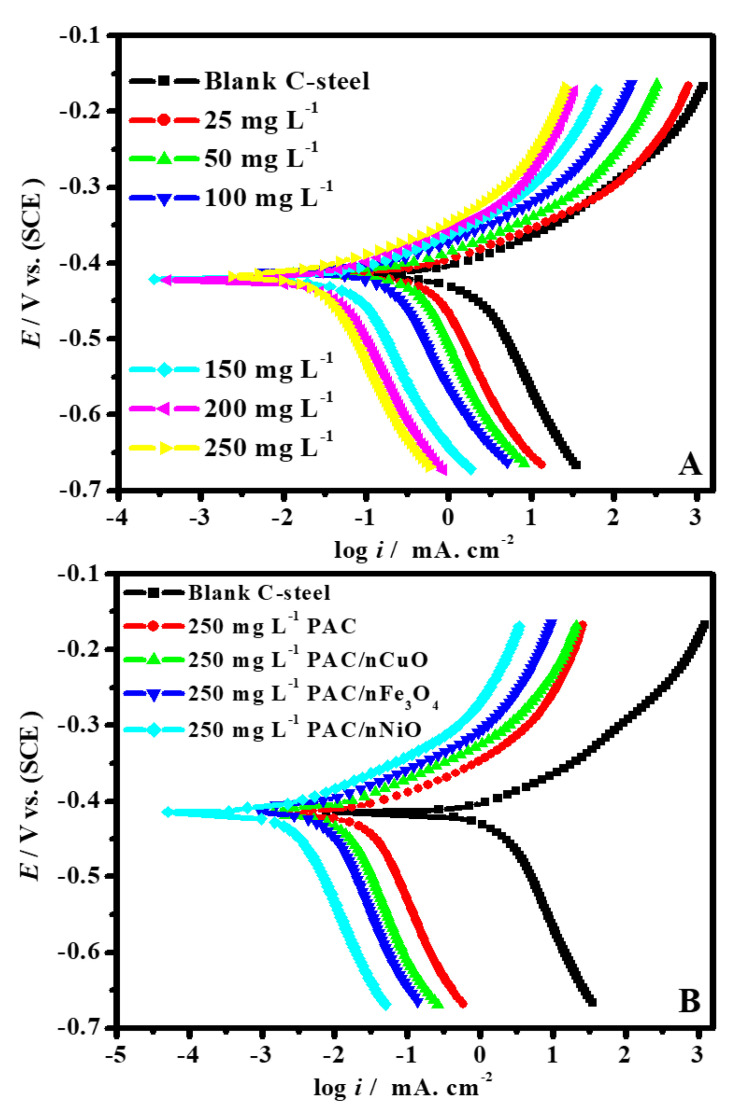
Tafel polarization profiles for C-steel in 1.0 M HCl solution without and with different concentrations of PAC (**A**) and in the presence of 250 mg L^−1^ of different nanocomposite inhibitors at 50 °C (**B**).

This test was accomplished to obtain information on the kinetics and the corrosion mitigation action of the as-synthesized nanocomposites on the C-steel substrate. The achieved PDP profiles for C-steel in 1.0 M hydrochloric acid in the absence and existence of various PAC and PAC/MONPs nanocomposite doses are displayed in Figure 7. Extrapolation of Tafel fit was applied to compute electrochemical indices including corrosion potential (*E*_cor_), corrosion current density (*i*_cor_), and anodic and cathodic Tafel slopes (*β*_c_ and *β*_a_). The consequences of PDP studies are documented in Table 1. The protection capacity (*η*_P_/%) and surface coverage (*θ*) were also intended based on Equation (2) [62] and the obtained results are displayed in Table 1:

(2) $$\eta_{P}/\%=\left(\frac{i_{\mathrm{cor}}^{0}-i_{\mathrm{cor}}^{i}}{i_{\mathrm{cor}}^{0}}\right)\times 100=\theta \times 100$$

where $i_{\mathrm{cor}}^{0}$ and $i_{\mathrm{cor}}^{i}$ are the values of *i*_cor_ in blank and inhibited medium, respectively. From Figure 7, both the anodic and cathodic branches minimized methodically with a rise in the dose of the PAC and PAC/MONPs nanocomposites, lacking much modification in the anodic and cathodic slopes. 

The modification in *E*_cor_ continued in between Δ*E* = ± 13 mV with respect to the uninhibited system; accordingly, the PAC and PAC/MONPs nanocomposites might be categorized as inhibitors of mixed-type [63,64,65,66,67,68,69,70,71,72,73,74,75]. When the PAC and PAC/MONPs nanocomposites were added to the corrosive solutions, the *i*_cor_ value was decreased from 2511 µA cm^−2^ to 298.8, 170.7, 97.9 and 35.1 µA cm^−2^ in the presence of higher dose (250 mg L^−1^) for PAC, PAC/CuONPs, PAC/Fe_3_O_4_NPs and PAC/NiONPs, respectively. These findings displayed that the fabricated PAC/MONPs nanocomposites can be applied as premium corrosion inhibitors for C-steel in hydrochloric acid solution, and the MONPs (Fe_3_O_4_, CuO and NiO) played a significant function in corrosion protection. Additionally, it is remarkable in Table 1 that the *η*_P_/% values of the four nanocomposites followed the order of *η*_P_ (PAC) > *η*_P_ (PAC/CuONPs) > *η*_P_ (PAC/Fe_3_O_4_NPs) > *η*_P_ (PAC/NiONPs), with the maximum protection capacity of 88.1%, 93.2%, 96.1% and 98.6%, respectively, and the investigational findings were in agreement with those achieved by the weight-loss method. The rise in *η*_P_/% with an increase in inhibitor dose could be associated with an increment in the number of adsorbed inhibitor species at a metal/solution surface, therefore leading to a rise in the surface coverage (*θ*) value. 

#### 3.2.3. EIS Studies 

**Figure 8:**
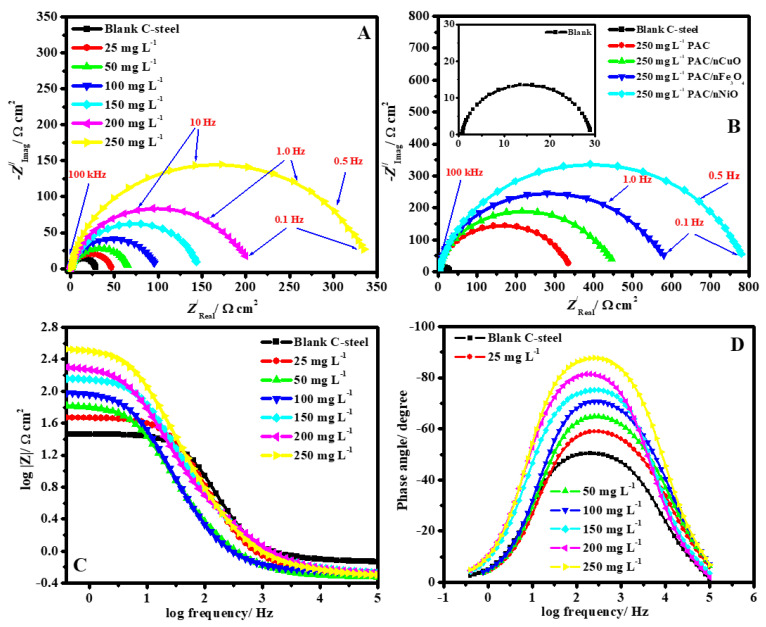
Impedance plots for C-steel in 1.0 M HCl solution; (**A**) Nyquist in the absence and presence of different concentration of PAC, (**B**) Nyquist in the absence and presence of 250 ppm of different nanocomposite inhibitors, (**C**) Bode and (**D**) phase angle representations in the absence and presence of different concentration of PAC at 50 °C.

In order to confirm the outcomes from the PDP and weight loss methods and fulfill more data about the mechanism of corrosion processes, the EIS investigation of C-steel in 1.0 M hydrochloric acid with different concentrations of PAC and PAC/MONPs nanocomposites was completed. Figure 8 represents the impedance plots for the working electrode in HCl solution: (A) Nyquist in the absence and existence of a various concentrations of PAC, (B) Nyquist in the absence and occurrence of 250 ppm of different nanocomposite inhibitors, (C) Bode and (D) phase angle representations in the absence and occurrence of a different concentration of PAC at 323 K. The Nyquist profiles obviously reveal that a rise in nanocomposites concentration (all individual PAC, PAC/nCuO, PAC/nFe_3_O_4_, and PAC/nNiO nanocomposites) leads to an attendant improvement in the capacitance loops diameter. The non-ideal half-circle feature of the Nyquist diagram could be elucidated in terms of surface inhomogeneity owing to the development of an adsorbed film of the as-prepared nanocomposite collected with the corrosion product [66]. 

The Nyquist profiles in the inhibited and uninhibited systems indicate the presence of a single capacitive loop and the lack of an inductive loop. The Bode diagram slope can deliver the ‘*n*’ parameter value (Table 2). It could be detected that the values of ‘*n*’ in the existence of PAC, PAC/CuO, PAC/Fe_3_O_4_, and PAC/NiO nanocomposites increased from 0.784 to 0.893 in comparison to the blank C-steel (0.701) specimen, which indicates that the C-steel interface became more homogeneous with the existence of PAC/CuO, PAC/Fe_3_O_4_, and PAC/NiO nanocomposites. In the middle frequency area of the phase angle profile (Figure 8D), the achieved values are within the range of −51.1° for the uninhibited C-steel to −87.5° with an increase in the PAC dose. In the circumstance of a perfect capacitor, the maxima phase angle in the middle frequencies scopes −90°. Thus, the phase angle approach towards the values of the angle −90 ° in the occurrence of PAC shows an improvement in the capacitive routine in the existence of the nanocomposites [67].

**Figure 9:**
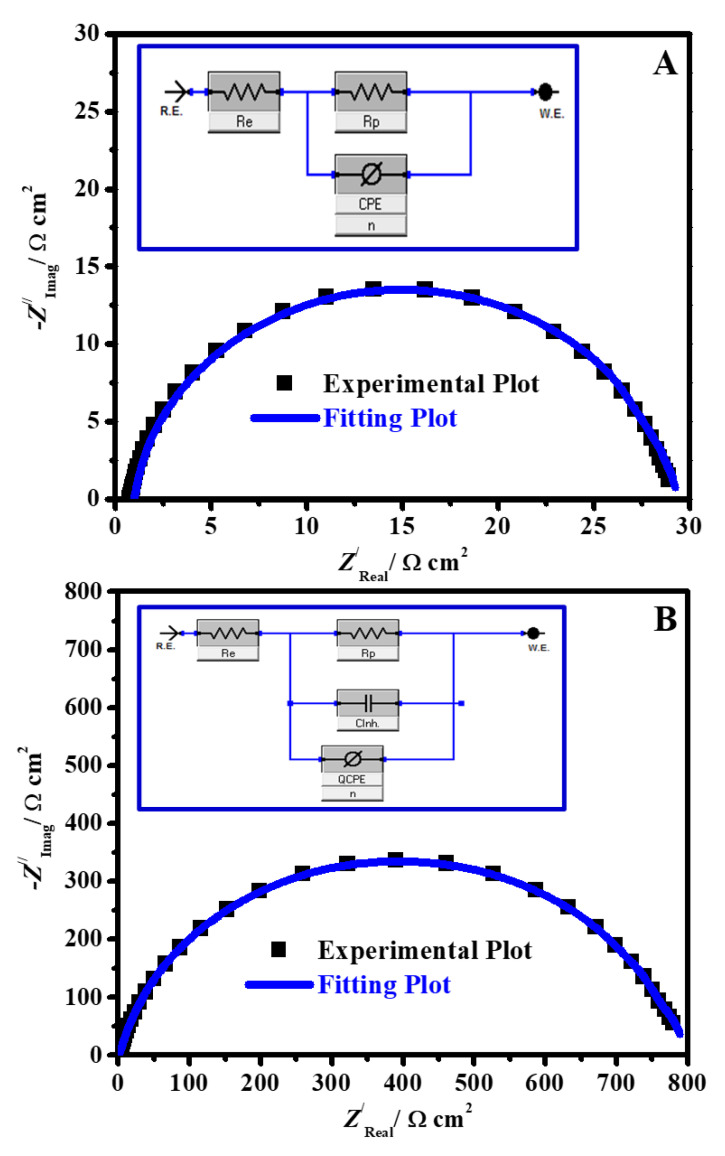
Experimental and fitted Nyquist results of C-steel in an uninhibited system (**A**) and an inhibited system (**B**) Inset equivalent circuits model for the phenomena.

The fitted EIS plots for the blank C-steel and inhibited system are displayed in Figure 9A,B. The impedance parameters recorded in Table 2 were obtained by fitting the accomplished EIS findings into the equivalent-circuit model for the phenomena depicted in Figure 9 insets (A, B) for uninhibited and inhibited systems, in which the electrolyte resistance (*R*_e_) is shorted by a CPE (constant phase element) in place of the classical double layer capacitor (*C*_dl_) that is in parallel to the resistance of polarization (*R*_p_); wherein, the *R*_p_ is equal to the *R*_ct_ (the resistance of charge transfer) + *R*_e_ (the electrolyte resistance) for the blank C-steel specimen as designated previously [68]. In the presence of inhibitors (inhibited medium), the *R*_p_ is the shared influence of the resistance from the *R*_ct_ + *R*_e_ + *R*_f_ (the layer resistance designed on the surface of C-steel), which is in series to the parallel of *C*_ads_ as capacitance because of the inhibitor adsorption layer [68]. The accuracy of the fitted EIS outcomes was appraised by the “fit goodness” chi-squared values (*χ*^2^). It could be seen from Table 2 that the *χ*^2^ values are very lesser, which (in the order of 10^−4^) boosts the appropriateness of the equivalent circuit model for precise reproduction of the impedance findings. The relation between impedance and CPE is specified as [69]:

(3) $$Z_{\mathrm{CPE}}={\left[Y_{0}{\left(j\omega \right)}^{n}\right]}^{-1}$$

where *j* characterizes the imaginary number, *Y*_0_ represents a proportionality coefficient, and *n* symbolize the phase shift. Regularly, CPE is transformed into the traditional endured elements resistance (*R*), capacitor (*C*), and inductance (*L*) when *n* = 0, 1, and −1, respectively. The protection capacity (*η*_E_/%) and surface coverage (*θ*) from the impedance data were computed from the following Equation [70]:

(4) $$\eta_{E}/\%=\left[\frac{R_{p}^{i}-R_{p}^{0}}{R_{p}^{i}}\right]\times 100=\theta \times 100$$

where $R_{p}^{0}$ and $R_{p}^{i}$ represent the *R*_p_ in the uninhibited and inhibited mediums, respectively. 

By inspection in Table 2, it was observed that the values of *R*_p_ increased intensely after the insertion of the as-prepared nanocomposites to the corrosive medium (i.e., 28.8 Ω cm^2^ vs. 353.9, 457.2, 614.4, 804.5 Ω cm^2^ in the presence of 250 mg L^−1^ PAC, PAC/CuONPs, PAC/Fe_3_O_4_NPs and PAC/NiONPs, respectively), demonstrating that these nanocomposites could efficiently impede the charge-transfer performance. Moreover, it should be indicated that the intended *η*_E_/% values of impedance spectroscopy increased with the nanocomposites addition, and, at the optimum dosage of 250 mg L^−1^ additives, the maximum protection power (*η*_E_) reached 91.8% for PAC, 93.7% for PAC/CuONPs, 95.3% for PAC/Fe_3_O_4_NPs and 96.4 % for PAC/NiONPs, respectively.

Furthermore, Table 2 exhibits that the double layer capacitance values (*C*_dl_) diminished regularly with an increment in nanocomposite dose. The degree of the decline in *C*_dl_ was larger for PAC/nNiO nanocomposite. The idea of electrochemical *C*_dl_ was first presented by Helmholtz and then explained by Geary and Stern [71]. Various parameters including surface area available to ions (*A*), the distance between the charged metal surface and the ion (*d*), the vacuum permittivity (*ε*_0_) and the electrolyte dielectric constant (*ε*_0_) determine the *C*_dl_, based on the following Equation [71]:

(5) Cdl=ε0εAd

An increase in composite dosage leads to a greater degree of *θ*, and thus decreases the efficacious value of *A*. Furthermore, the nanocomposite species (PAC/MONPs) are adsorbed at the interface of steel/HCl, substituting water molecules and pre-adsorbed Cl^−^ ions. This shrinks the solution dielectric-constant of the abutment to the steel substrate. Meanwhile, the pre-adsorbed Cl^−^ ions create the double layer prevalent away from the steel interface, the active double layer thickness rises [72]. All these features act accommodatingly in dropping the electrochemical *C*_dl_, per unit surface-area in the occurrence of PAC/MONPs additives. The different rates of corrosion-decreasing impacts were more predominant for PAC/NiO nanocomposite, determining it the superior inhibitor. This is also apparent from the lesser *C*_dl_ and greater *R*_p_ values for PAC/NiONPs compared to that for PAC, PAC/CuONPs, and PAC/Fe_3_O_4_NPs at the same conditions.

### 3.3. Adsorption Consedrations 

The inhibitors diminish the C-steel corrosion rate by adsorption at the electrode/medium surface, whereas the significant indices of interaction between the steel interface and inhibitor molecule could be achieved from the models of adsorption isotherm [60]. Inhibitor structure, metal nature and surface charge, corrosive solution type, and the distribution of molecular charge are aspects influencing inhibitors in adsorption routes [3]. With an increment in the investigated nanocomposite dose, the protection capacity is augmented. This point could be clarified by the adsorption of these nanocomposites on the C-steel interface. In the current report, the *θ* values were endeavored to be fitted into various isotherm models such as Temkin, Flory–Huggins, Langmuir, and Frumkin. 

**Figure 10:**
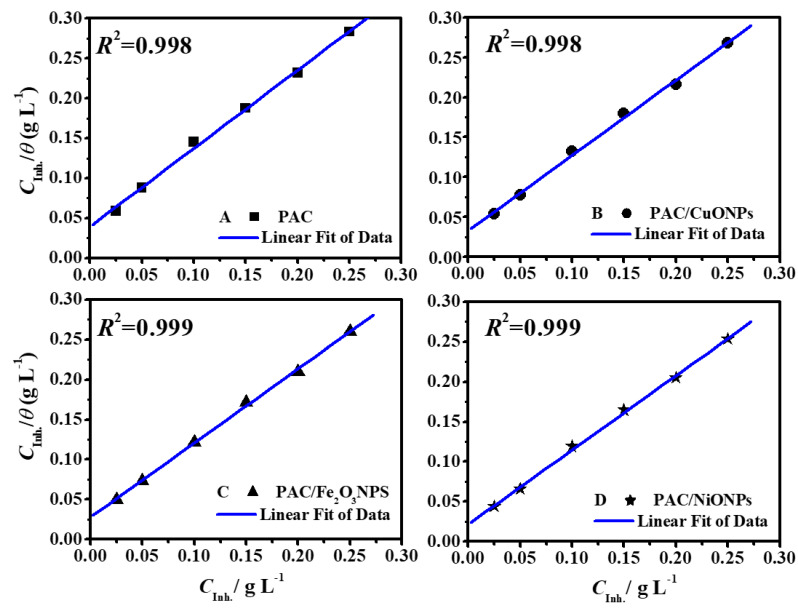
Langmuir profile of C/*θ* vs. C from potentiodynamic polarization (PDP) findings for C-steel in 1.0 M HCl solution in the presence of (**A**) PAC, (**B**) PAC/CuONPs, (**C**) PAC/Fe_3_O_4_NPs, and (**D**) PAC/NiONPs at 50 °C.

Adsorption isotherms of PAC, PAC/CuO, PAC/Fe_3_O_4_ and PAC/NiO nanocomposites on C-steel by their *θ* values as a function of PAC/MONPs dose in molar hydrochloric acid solution (*C*_inh_) are displayed in Figure 10. The diagrams designate that all PAC, PAC/nCuO, PAC/nFe_3_O_4_ and PAC/nNiO nanocomposites follow a linear Langmuir model inclination, as straightforward lines were produced with regression coefficient values (*R*^2^) ~1 (Table 3). The Langmuir model is specified by the next formula [4]:

(6) $$\frac{C_{\mathrm{Inh}}}{\theta }=C_{\mathrm{Inh}}+\frac{1}{K_{\mathrm{ads}}.}$$

Herein, *K*_ads_ symbolizes the adsorption equilibrium constant and *θ* is measured from PDP studies. The *K*_ads_ is connected with the standard free energy of adsorption ($\Delta G_{\mathrm{ads}}^{0}$), through Equation [5]:

(7) $$\Delta G_{\mathrm{ads}}^{0}=-RT\ln(1\times 10^{6}K_{\mathrm{ads}})$$

where, *R* = universal gas constant, *T* = absolute temperature, and the factor 1 × 10^6^ is [H_2_O] stated in g L^−1^. Anti-corrosive additives are supposed to adsorb at a C-steel/medium interface with a sequences of protuberant binding centers. As all centers are conformable, so each center could connect to only one inhibitor.

The obtained thermodynamic indices from the Langmuir adsorption model for the as-fabricated nanocomposites are recorded in Table 3. It is noticeable from the findings that high *K*_ads_ values indicate the powerful interaction between the investigated PAC/nMONPs and metal surfaces. Recently, it was found that high values of *K*_ads_ touch on more powerful and steadier adsorbed film development on the C-steel interface [62]. In general, a value of $\Delta G_{\mathrm{ads}}^{0}$ higher than or around −40.0 kJ mol^−1^ (more negative) comprises charge sharing between the C-steel and nanocomposites (chemical adsorption) [63]. In the present work, the computed $\Delta G_{\mathrm{ads}}^{0}$ values for as-prepared nanocomposites PAC, PAC/CuONPs, PAC/Fe_3_O_4_NPs and PAC/NiONPs are −45.82, −46.26, −46.79 and −47.34 kJ g^−1^, respectively. This is a suggestion of predominant chemical adsorption, but the physical adsorption contribution could also be measured owing to the opportunity of development of protonated compound which could interact electrostatically with a charged steel surface.

### 3.4. Comparative Study with Some Reported Inhibitors

We have compared the protection capacity of the as-prepared PAC, PAC/CuONPs, PAC/Fe_3_O_4_NPs, and PAC/NiONPs intended from the potentiodynamic polarization studies with the formerly reported additives on the analogous substrate. A comparative investigation of our obtained outcomes with analogous studies [65,73,74,75] has been recorded in Table 4. With noteworthy protection for C-steel in acidic pickling solutions (HCl) at a dose as low as 250 mg L^−1^ for PAC, PAC/CuO, PAC/Fe_3_O_4_ and PAC/NiO nanocomposites could serve as anticorrosive inhibitors for C-steel in an acidic environment as the marketable additives with practical industrial application. 

### 3.5. Surface Morphology 

#### 3.5.1. FE-SEM and EDX Analysis 

**Figure 11:**
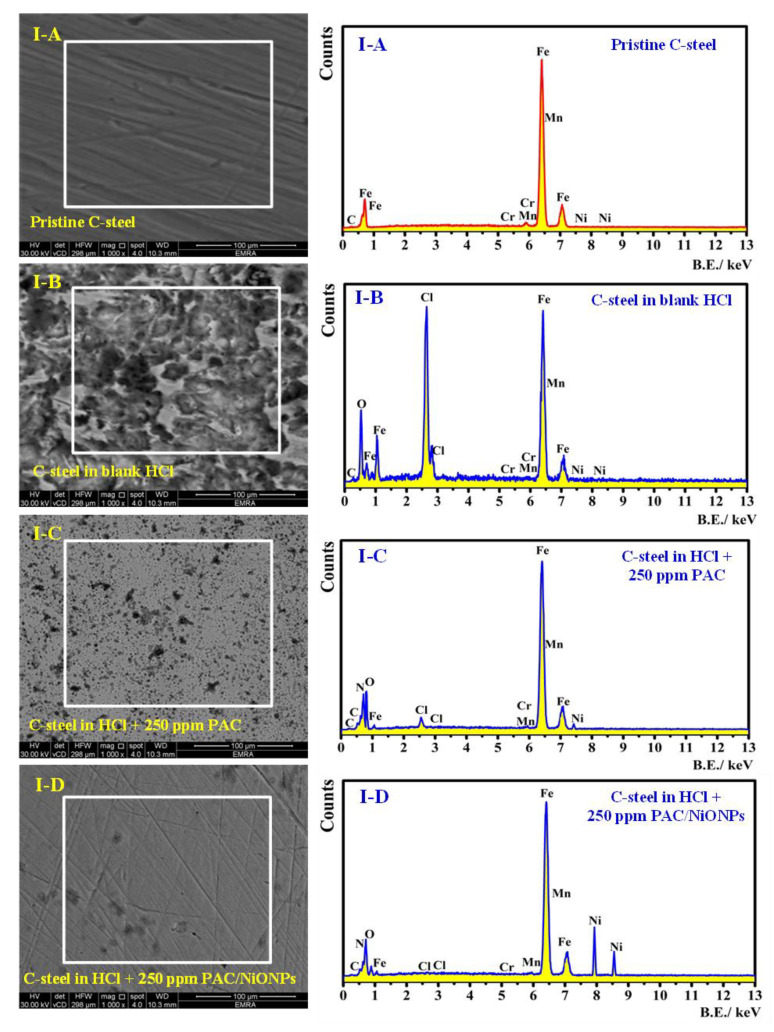
FE-SEM (I) and EDX (II) analyses for (**A**) pristine C-steel, (**B**) corroded C-steel in blank HCl, (**C**) C-steel with the occurrence of 250 ppm PAC and (**D**) C-steel with the presence of 250 ppm PAC/NiONPs.

The surface topology of C-steel owing to the process of corrosion was inspected under FE-SEM micrographs accompanied by EDX to measure the elemental arrangements on the metal interface before and after immersion in the corrosive medium in the lack and the occurrence of as-prepared nanocomposites. Figure 11A–D shows an arrangement of the FE-SEM (I) and EDX (II) analyses for (A) polished C-steel, (B) corroded metal in HCl free inhibitor, (C) C-steel with the occurrence of 250 ppm PAC, and (D) C-steel with the presence of 250 ppm PAC/NiONPs. No signal of corrosion accomplishment has occurred on the pristine C-steel surface presented in Figure 11A. 

Figure 11B exposed that, without any inhibitors (blank medium 1.0 M HCl), the C-steel surface looked to be extremely damaged with parts of restricted corrosion. Nevertheless, in the solution containing 250 mg L^−1^ PAC in Figure 11C, the corrosion action was repressed as observed from the decline in restricted corrosion zones. This is related to the PAC additive adsorption at the metal/solution interface creating a mono-film of protection vs. corrosion action. A smoother C-steel surface could be detected from Figure 11D in the inhibited system containing 250 mg/L PAC/NiONPs. This elucidates a more reduction in the corrosion activity and might be owing to the co-adsorption of PAC and NiONPs species on the metal surface, consequently advancing inhibition capacity.

From EDX analysis in Figure 11II and Table 5, elemental investigation of the corroded specimen, Figure 11B(II) using EDX indicated that the percentage of Cl and O are largely owing to the development of corrosion film from adsorptive ferrous-chloride (FeCl)_ads_, which could promote corrosion to produce FeOOH and Fe(OH)_3_ [76]. In the presence of an aggressive medium containing 250 mg L^−1^ PAC, only with a quantity of chloride and oxygen was it noticed demonstrating that the corrosion activity had been repressed considerably, in addition to the appearance of a new peak for N atoms. The occurrence of PAC as an additive reduced the O reduction route, therefore, decreasing the rate of corrosion. The declined O value displays the inhibitor activity [77].

PAC/NiONPs showed a positive effect in prohibiting the C-steel corrosion as displayed in the FE-SEM micrograph (Figure 11D). The Fe% is more than that of the corroded specimen with only less than 13% elements of O and Cl. Additional negative charge produced from the Cl^−^ adsorption enhances interaction by electrostatic with protonated PAC/NiONPs (due to the presence of primary -NH_2_) (cation) [78]. Interaction by electrostatic was supposed to be produced between (FeCl^−^)_ads_ species at anodic positions and the protonated molecules [79]. Surface morphology using FE-SEM demonstrated a substantial enhancement on the interface of the C-steel substrates in the existence of an optimum dose of 250 ppm of PAC and PAC/NiONPs inhibitors.

#### 3.5.2. FI-TR Analysis 

**Figure 12:**
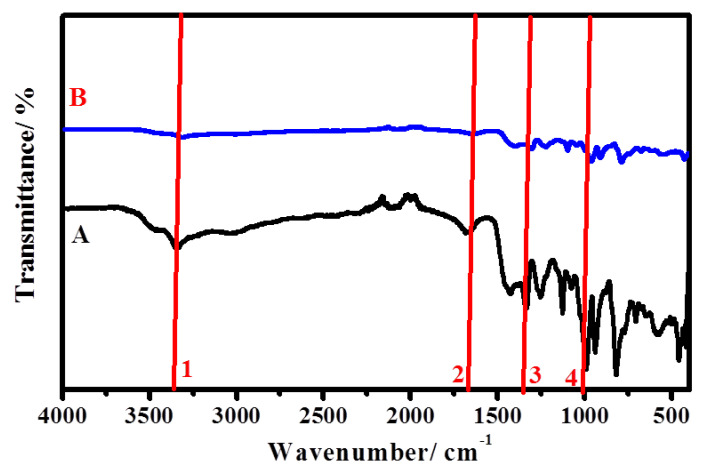
FT-IR spectra of (**A**) crude PAC/NiONPs inhibitor, (**B**) adsorbed film formed on the C-steel surface after dipping in 1.0 M HCl containing 250 ppm PAC/NiONPs.

Herein, FT-IR examinations were used to confirm that the corrosion inhibition of C-steel in the investigated corrosive medium is associated with the inhibitor adsorption on the metallic interface. In FT-IR exploration of PAC/NiONPs inhibitor (line A), the adsorbed film formed on the C-steel surface after dipping in molar HCl containing 250 ppm PAC/NiONPs (line B) is presented in Figure 12. PAC/NiONPs compound has diverse effective groups that possess an electron cloud. This designates that the adsorption layer between the prepared PAC/NiONPs inhibitor and empty d-orbitals of Fe is probable to occur within these well-designed groups. The FT-IR feature of PAC/NiONPs and the film formed on the electrode interface via adsorption were reasonably clarified. The broad peak (peak 1) at near 3340 cm^−1^ is related to the -OH in PAC/NiONPs. This broadband (peak 1; Figure 12B) disappeared in the case of an inhibited system (adsorbed layer), which could be ascribed to the chemical bond formed at the C-steel surface (Figure 12B). Furthermore, bands 2 and 3 (Figure 12A) at 1669 cm^−1^ and 1332 cm^−1^ related to the -C-N- peak, the frequency absorption bands altered to 1639 cm^−1^ and 1484 cm^−1^ with PAC/NiONPs (Figure 12B). The modifications in absorption bands display the adsorption of diverse functional groups of the PAC/NiONPs at the interface of the metal/electrolyte and the formation of [Fe PAC/NiONPs] on the metallic interface [80]. Mourya et al. specified that the variation in peaks is either because of adsorption, or due to optical effects, or owing to chemical reactions with the electrolyte.

### 3.6. The Corrosion Mitigation Mechanism by Primary Aminated Modified Cellulose (PAC) Containing Nano-Oxide of Some Metals (MONPs)

From the attained data in this study, individual PAC has reasonable protection efficacy on C-steel in molar HCl medium. From Table 1, the highest protection capacity in the existence of 250 ppm PAC is 88.1% at 50 °C from PDP experiments. The value of $\Delta G_{\mathrm{ads}}^{0}$ displayed that the PAC adsorption at the metal/electrolyte interface involves predominant chemical adsorption, but the physical adsorption also contributed. In the studied acidic solution, PAC polymer was primarily present in PACH^+^ (protonated structure), and when the C-steel surface attained positive charge, consequently, the metal surface was hydrated with Cl^−^ ions, and the PAC protonated compound could interact electrostatically with a negatively charged steel surface (physical adsorption).

As well as the physical adsorption, there should be chemisorption attributed to the coordinate bonds that might be designed among vacant d-orbitals of Fe surface and efficient groups in PAC molecules. The achieved outcomes in the present study display that the protection efficacy and stability of PAC have been significantly improved by the incorporation of Fe_3_O_4_, CuO, and NiO NPs in the PAC matrix. For instance, 250 ppm PAC/Fe_3_O_4_NPs, PAC/CuONPs, and PAC/NiONPs in molar HCl medium at 50 °C reveal inhibition efficiency of 93.2, 96.1 and 98.6%, respectively. Remarkably, when the metal electrode was dipped in HCl containing PAC/Fe_3_O_4_NPs, PAC/CuONPs, and PAC/NiONPs nanocomposites, the cationic form of additive species first adsorbed at the electrode/electrolyte surface via physical adsorption between the protonated PAC/Fe_3_O_4_NPs, PAC/CuONPs, and PAC/NiONPs molecules and the Cl^−^ ions hydrated interface. It appears that attractions occur among the iron-atoms on the steel metal and Fe_3_O_4_, CuO, and NiO NPs as a result of the efficient characteristics of the Fe_3_O_4_, CuO, and NiO NPs [64]. The hetero-atoms in PAC have a lone pair of electrons that could be transferred to the vacant d-orbital of Fe. The existence of MONPs (Fe_3_O_4_NPs, CuONPs and NiONPs) in the PAC construction may have also prohibited the polymer torsion [65], which leads to total coverage of the metal interface. 

## 4. Conclusions

In this study, primary aminated cellulose (PAC) and the in-situ deposition of some metal oxide nanoparticles (MONPs) in PAC matrix were prepared, and their structures were characterized through FT-IR, FE-SEM/EDX, TEM, SAED, and XRD. For the first time, PAC, PAC/Fe_3_O_4_NPs, PAC/CuONPs, and PAC/NiONPs exhibited superior inhibitive action for C-steel in acidic chloride solutions. The protection capacities of these as-prepared nanocomposites by electrochemical approaches and weight-loss experiments followed the order of PAC/NiONPs (98.6%) > PAC/Fe_3_O_4_NPs (96.1%) > PAC/CuONPs (93.2%) > PAC (88.1%). The PDP profiles demonstrated that the synthesized nanocomposites act as the inhibitors of mixed-type, whereas the intended thermodynamic parameters specified chemisorption as the main adsorption that follows the Langmuir isotherm model. The surface topology was evaluated by FT-IR, FE-SEM, and EDX analysis, and established the adsorption of PAC, PAC/Fe_3_O_4_NPs, PAC/CuONPs, and PAC/NiONPs molecules at the metal/HCl interface and that they accordingly played a role in the worthy corrosion protection activity. Therefore, it could be decided that PAC and PAC/MONPs are respectable eco-friendly inhibitors for steel corrosion in pickling HCl solutions.

## Figures and Tables

**Table 1:** PDP parameters for C-steel in molar hydrochloric acid without and with different concentrations of the as-prepared nanocomposite inhibitors at 50 °C.

Inhibitor Code	*C*_inh_/ mg L^−1^	*i*_cor_ ± SD/ µA cm^−2^	*−E*_cor_/ V (SCE)	*β*_a_/ V dec^−1^	*−β*_c_/ V dec^−1^	*θ*	*η*_P_/ %
Blank	0.0	2511 ± 195	−0.416	0.081	0.161	--	--
**PAC**	25	1453.8 ± 122	−0.414	0.088	0.176	0.421	42.1
	50	1089.7 ± 105	−0.412	0.093	0.176	0.566	56.6
	100	785.9 ± 65	−0.411	0.095	0.173	0.687	68.7
	150	509.7 ± 43	−0.426	0.084	0.174	0.797	79.7
	200	344.1 ± 23	−0.425	0.087	0.178	0.863	86.3
	250	298.8 ± 11	−0.418	0.091	0.18	0.881	88.1
**PAC/CuONPs**	25	1345.8 ± 113	−0.410	0.095	0.179	0.464	46.4
	50	906.4 ± 86	−0.405	0.092	0.182	0.639	63.9
	100	617.7 ± 56	−0.425	0.089	0.176	0.754	75.4
	150	414.3 ± 34	−0.420	0.094	0.182	0.835	83.5
	200	193.3 ± 16	−0.422	0.087	0.185	0.923	92.3
	250	170.7 ± 10	−0.416	0.091	0.181	0.932	93.2
**PAC/Fe_3_O_4_NPs**	25	1247.9 ± 102	−0.403	0.095	0.174	0.503	50.3
	50	806.1 ± 77	−0.423	0.087	0.178	0.679	67.9
	100	441.9 ± 41	−0.430	0.092	0.176	0.824	82.4
	150	313.8 ± 26	−0.410	0.092	0.174	0.875	87.5
	200	118.1 ± 12	0.407	0.089	0.177	0.953	95.3
	250	97.9 ± 8	−0.412	0.093	0.183	0.961	96.1
**PAC/NiONPs**	25	1084.7 ± 97	−0.425	0.093	0.174	0.568	56.8
	50	612.6 ± 51	−0.432	0.094	0.181	0.756	75.6
	100	404.2 ± 37	−0.413	0.095	0.18	0.839	83.9
	150	223.5 ± 18	−0.408	0.088	0.181	0.911	91.1
	200	67.8 ± 6	−0.411	0.091	0.176	0.973	97.3
	250	35.1 ± 3	−0.415	0.093	0.179	0.986	98.6

**Table 2:** Impedance parameters for C-steel in molar hydrochloric acid without and with different concentrations of the as-prepared nanocomposite inhibitors at 50 °C.

Inhibitor Codes	*C*_inh_/ mg L^−1^	*R*_e_/ Ω cm^2^	*R*_P_/ Ω cm^2^ ± SD	*C*_dl_/ F cm^−2^ × 10^−6^	*Q*_CPE_		*χ*^2^ × 10^−4^	*θ*	*η*_E_/ %
					*Y*_0_/μΩ^−1^ s^n^ cm^−2^	*n*			
**1.0 M HCl**	**0.0**	**0.69**	**28.8 ± 2.1**	**822.5**	**75.84**	**0.701**	**4.52**	**--**	**--**
PAC	25	0.78	48.2 ± 3.2	310.8	27.22	0.784	3.72	0.402	40.2
	50	0.73	69.1 ± 4.3	251.6	22.24	0.793	3.77	0.583	58.3
	100	0.71	101.4 ± 7.3	140.8	12.39	0.872	3.81	0.715	71.5
	150	0.89	150.3 ± 11.5	92.5	8.02	0.812	3.86	0.808	80.8
	200	0.82	213.6 ± 15.6	81.6	6.44	0.862	4.01	0.865	86.5
	250	0.98	353.9 ± 21.7	56.6	4.98	0.822	3.90	0.918	91.8
PAC/CuONPs	25	0.85	49.8 ± 3.1	245.8	21.51	0.793	3.77	0.422	42.2
	50	0.81	74.2 ± 5.2	204.1	17.86	0.784	3.79	0.612	61.2
	100	0.78	115.6 ± 7.8	128.3	11.30	0.803	3.81	0.751	75.1
	150	0.97	189.4 ± 9.5	68.3	5.95	0.812	3.94	0.848	84.8
	200	0.93	313.1 ± 15.7	54.1	3.76	0.889	3.97	0.908	90.8
	250	1.07	457.2 ± 24.3	40.8	3.52	0.829	3.86	0.937	93.7
PAC/Fe_3_O_4_NPs	25	0.81	51.7 ± 3.9	185.2	16.16	0.812	4.19	0.443	44.3
	50	0.84	82.7 ± 5.7	126.6	11.06	0.825	4.14	0.652	65.2
	100	0.89	142.5 ± 8.4	103.3	8.99	0.812	3.97	0.798	79.8
	150	0.94	223.2 ± 12.4	55.8	6.56	0.822	3.55	0.871	87.1
	200	1.02	374.1 ± 18.9	42.5	4.98	0.847	3.69	0.923	92.3
	250	1.12	614.4 ± 27.5	30.1	2.67	0.851	4.04	0.953	95.3
PAC/NiONPs	25	0.85	55.9 ± 4.3	152.5	11.18	0.852	4.26	0.485	48.5
	50	0.87	97.3 ± 6.2	104.1	7.65	0.866	4.22	0.704	70.4
	100	0.93	176.6 ± 13.3	85.3	6.19	0.852	4.04	0.837	83.7
	150	0.98	271.7 ± 17.2	45.8	4.49	0.863	3.61	0.894	89.4
	200	1.06	564.7 ± 25.9	35.2	3.40	0.889	3.76	0.949	94.9
	250	1.17	804.5 ± 56.4	24.1	1.82	0.893	4.12	0.964	96.4

**Table 3:** Adsorption parameters derived from Langmuir model for C-steel in molar HCl for PAC /MONPs at 50 °C using PDP outcomes.

Inhibitor	*R* ^2^	*S* = slope	*K*_ads_ ( L g^−1^)	$\Delta G_{\mathrm{ads}}^{0}$/kJ g^−1^
**PAC**	0.998	0.978	25.65	−45.82
**PAC/CuONPs**	0.998	0.942	30.30	−46.26
**PAC/Fe_2_O_3_NPs**	0.999	0.929	37.03	−46.79
**PAC/NiONPs**	0.999	0.932	45.45	−47.34

**Table 4:** Comparison of the protection capacity of synthesized nanocomposites with existing literature.

Substrate	Corrosive Media	Inhibitor/Dose	Protection Capacity	References
St37 Steel	15% H_2_SO_4_	1000 ppm carboxymethyl cellulose/ silver nanoparticles composite	92.12	Ref. [65]
X60 pipeline steel	3.5% NaCl saturated with CO_2_	100 ppm chitosan	45	Ref. [73]
X60 pipeline steel	3.5% NaCl saturated with CO_2_	100 ppm carboxymethyl cellulose	39	Ref. [73]
X60 pipeline steel	3.5% NaCl saturated with CO_2_	100 ppm commercial inhibitor	88	Ref. [73]
Mild steel	2.0 M H_2_SO_4_	500 ppm carboxymethyl cellulose	66.2	Ref. [74]
Mild steel	1.0 M HCl	700 ppm sodium carboxymethyl cellulose	86.8	Ref. [75]
C-steel	Molar HCl	250 ppm PAC	88.1	Present work
C-steel	Molar HCl	250 ppm PAC/CuONPs	93.2	Present work
C-steel	Molar HCl	250 ppm PAC/Fe_3_O_4_NPs	96.1	Present work
C-steel	Molar HCl	250 ppm PAC/NiONPs	98.6	Present work

**Table 5:** The element percentage for each sample obtained from the EDX analysis after 48 h immersion in molar hydrochloric acid.

Specimen	Element (% wt)							
	Fe	C	Cr	Mn	Ni	O	N	Cl
(A) Pristine C-steel	98.94	0.19	0.75	0.06	0.06	--	--	--
(B) C-steel in molar HCl	61.34	0.18	0.61	0.05	0.02	16.15	--	21.65
(C) C-steel in molar HCl + 250 ppm PAC	86.12	4.67	0.23	0.03	0.05	4.37	2.37	2.21
(D) C-steel in molar HCl + 250 ppm PAC/NiONPs	87.25	3.04	0.16	0.04	3.16	3.08	1.87	1.40

## Data Availability

The raw/processed data generated in this work are available upon request from the corresponding author.
